# Identification of staphylococcal phage with reduced transcription in human blood through transcriptome sequencing

**DOI:** 10.3389/fmicb.2015.00216

**Published:** 2015-03-24

**Authors:** Tasha M. Santiago-Rodriguez, Mayuri Naidu, Marcus B. Jones, Melissa Ly, David T. Pride

**Affiliations:** ^1^Department of Pathology, University of CaliforniaSan Diego, CA, USA; ^2^J. Craig Venter InstituteRockville, MD, USA; ^3^Department of Medicine, University of CaliforniaSan Diego, CA, USA

**Keywords:** *Staphylococcus aureus*, transcriptome, RNA Seq, human microbiome, bacteriophage, prophage, mobile genetic element

## Abstract

Many pathogenic bacteria have bacteriophage and other mobile genetic elements whose activity during human infections has not been evaluated. We investigated the gene expression patterns in human subjects with invasive Methicillin Resistant *Staphylococcus aureus* (MRSA) infections to determine the gene expression of bacteriophage and other mobile genetic elements. We developed an *ex vivo* technique that involved direct inoculation of blood from subjects with invasive bloodstream infections into culture media to reduce any potential laboratory adaptation. We compared *ex vivo* to *in vitro* profiles from 10 human subjects to determine MRSA gene expression in blood. Using RNA sequencing, we found that there were distinct and significant differences between *ex vivo* and *in vitro* MRSA gene expression profiles. Among the major differences between *ex vivo* and *in vitro* gene expression were virulence/disease/defense and mobile elements. While transposons were expressed at higher levels *ex vivo*, lysogenic bacteriophage had significantly higher *in vitro* expression. Five subjects had MRSA with bacteriophage that were inhibited by the presence of blood in the media, supporting that the lysogeny state was preferred in human blood. Some of the phage produced also had reduced infectivity, further supporting that phage were inhibited by blood. By comparing the gene expression cultured in media with and without the blood of patients, we gain insights into the specific adaptations made by MRSA and its bacteriophage to life in the human bloodstream.

## Introduction

*Staphylococcus aureus* is a pathogen that is also considered normal human flora, and often takes advantage of breaks in protective skin barriers to cause disease (Chaffin et al., 2012). While *S. aureus* strains were primarily treatable with beta-lactam antibiotics in the past, their widespread use has resulted in the emergence of Methicillin-Resistant *S. aureus* (MRSA) strains. MRSA can be acquired in hospital- or community-based settings, but many of the Community-Acquired MRSA (CA-MRSA) strains have replaced more traditional Hospital-Acquired (HA-MRSA) strains in both environments (Shopsin et al., 2003; Popovich et al., 2008; Kennedy et al., 2010). CA-MRSA generally has been responsible for many invasive soft tissue infections, and has several virulence factors including Panton-Valentine leukocidin (PVL), which are thought to contribute greatly to its pathogenesis. PVL, which is not regularly detected in Hospital Acquired (HA)-MRSA strains, have been associated with epidemics of several CA-MRSA strains in the United States (Pan et al., 2003; Vandenesch et al., 2003). Virulence factors are of particular interest in invasive bloodstream infections, and many are derived from mobile genetic elements, including plasmids, bacteriophage, transposons, and pathogenicity islands (Bae et al., 2006; Baba et al., 2008; Diep et al., 2008b).

There usually are bacteriophage integrated into the genomes of *S. aureus* isolates, with most of them belong to the phage family Siphoviridae (Canchaya et al., 2003; Feng et al., 2008). These phage are of intermediate size (generally around 40 to 45 kb) and often carry toxins that may contribute to pathogenesis (Brussow et al., 2004). Some phage carry the immune modulator staphylokinase, which is responsible for host tissue destruction. Others encode toxins such as PVL, or superantigens involved in toxic shock syndrome, necrotizing fasciitis, and food poisoning (Deghorain and Van Melderen, 2012). These phage likely impact staphylococcal pathogenesis through lysogenic conversions, where the virulence functions they carry are expressed during infection in humans. The expression of these phage may directly reflect their contributions to pathogenesis, but has not been thoroughly examined during human bloodstream infections.

The expression of genes involved in the pathogenicity of CA-MRSA has been mainly studied *in vitro* (Cui et al., 2005; Lindsay et al., 2006; Stevens et al., 2007; Pohl et al., 2009). Few studies have compared the *in vivo* and *in vitro* gene expression of MRSA, and generally have been restricted to animal models (Diep et al., 2008a,b; Chaffin et al., 2012), which may not fully reflect the adaptive behavior of the pathogen in humans. Other *ex vivo* strategies have grown a single, lab-adapted MRSA strain in the presence of healthy human donor blood to characterize virulence genes that may be overexpressed (Malachowa and Deleo, 2011; Malachowa et al., 2011). No studies, however, have studied MRSA across human subjects with varying degrees of illnesses to understand whether the behavior of MRSA and their mobile genetic elements is reproducible across different human subjects. It is of substantial importance to understand the contributions of phage and other mobile genetic elements to MRSA pathogenesis during human infections and their potential for spread to others.

The primary tool for analysis of global patterns of gene expression in microbes is transcriptomics. While some have studied transcriptomics in MRSA utilizing quantitative PCR (Sabersheikh and Saunders, 2004), or microarray analysis (Witney et al., 2005; Lindsay et al., 2006), RNA sequencing provides a tool for characterization of patterns of gene expression that does not require a priori information about the pathogen being studied (Wilhelm and Landry, 2009). Thus, gene expression patterns from RNA sequencing can be used to thoroughly characterize novel MRSA strains. RNA sequencing technology has not been employed to characterize global changes in *S. aureus* gene expression in humans with invasive bloodstream infections. Here, we report the RNA sequencing expression profiles of MRSA strains grown in the presence of blood from 10 human subjects with invasive bloodstream infections and characterize differences observed in the expression of bacteriophage and other mobile genetic elements by comparing expression profiles with and without human blood.

## Results

### Human subjects and RNA enrichment

We sampled blood from 10 human subjects with invasive bloodstream MRSA infections (Table 1 and Supplemental Table 1), and cultured each to further our understanding of the gene expression of MRSA in the human bloodstream. We utilized an *ex vivo* technique that involved direct inoculation at the bedside of blood from human subjects with invasive bloodstream infections into culture media that bypassed the need for a separate *in vitro* culture step; thus, the isolates from each subject were characterized directly from blood with minimal opportunity for gene expression changes that might accompany laboratory adaptation. The cohort of subjects in this study included many that were critically-ill (Table 1), which significantly contrasts with a prior study characterizing a single lab-adapted MRSA strain cultured in the presence of blood from healthy donors (Malachowa et al., 2011). For comparison, we performed a separate isolation of each MRSA strain, and cultured each *in vitro* to help decipher comparatively those genes whose expression might be induced through exposure to the human bloodstream. Samples 21MRA and 23MRA were isolated from the same individual 48 h apart, and were utilized to help determine whether results would be consistent within individual subjects over time. All *ex vivo* and *in vitro* MRSA isolates were grown to log phase (Supplemental Figure 1), and total RNA was isolated from each subject/MRSA isolate under both growth conditions. Because the *ex vivo* samples were cultured in the presence of blood from each human subject, we enriched to remove any RNA that may have been derived from the human host. The resulting RNA was sequenced from all 10 subjects for a total of 6,213,492 *ex vivo* reads (mean of 564,863 ± 79,540 per subject) and 4,568,420 *in vitro* reads (mean of 415,310 ± 10,582 per subject) (Table 2). The inclusion of the enrichment step substantially reduced the presence of human RNA, as exemplified by the relatively low percentage of RNA identified that was homologous to human DNA (mean 1.22 ± 0.55%; range from 0.17% to 6.26%). All sequence reads homologous to human DNA were removed prior to further analysis.

**Table 1:** **Study subjects**.

	**Age**	**Sex**	**Diagnosis**	**Comorbidities**	**Antibiotics^a^**
1MRA	64	Male	Bacteremia; severe sepsis; abscess	Quadriplegia; hypertension	Vancomycin/Gentamicin/Rifampin
3MRA	57	Female	Bacteremia; sepsis	Metastatic colon cancer	Vancomycin
4MRA	84	Female	Bacteremia; sepsis; infected dialysis graft	End-stage renal disease	Vancomycin/Gentamicin/Televancin
6MRA	64	Female	Bacteremia; septic arthritis	Metastatic colon cancer	Vancomycin
20MRA	60	Male	Bacteremia; sepsis; furunculosis	Asthma	Vancomycin
21/23MRA^b^	50	Male	Bacteremia; sepsis; line infection	Dermatomyositis	Vancomycin/Daptomycin
31MRA	60	Female	Bacteremia; severe sepsis	Schleroderma	Vancomycin/Piperacillin/Tazobactam
55MRA	60	Female	Bacteremia; sepsis; line infection	Congestive heart failure	Vancomycin/Rifampin
74MRA	81	Male	Bacteremia; sepsis; Pneumonia	High blood pressure	Vancomycin/Piperacillin/Tazobactam
256MRA	73	Female	Bacteremia; sepsis; osteomyelitis	T12 paraplegia; sacral decubitus ulcer	Vancomycin/Piperacillin/Tazobactam

a *Antibiotics administered within 24 h prior to the sample collection*.

b *Subjects 21 and 23 represent the same individual for which samples were collected at different time points*.

**Table 2:** **RNA sequences from all subjects**.

	**Reads**	**Average length**	**Homologous to human genome**	**After trimming**	**Reads mapping to *S.aureus* (%)**	**Top genome**
***EX VIVO***						
1 MRA	505,449	103	27,413	437,829	427,847 (97.72)	*S. aureus* COL
3 MRA	1,266,504	106	3447	1,166,335	1,164,602 (99.85)	*S. aureus* COL
4 MRA	1,023,554	105	490	880,026	878,868 (99.87)	*S. aureus* JH1
6 MRA	630,865	104	11,848	575,675	571,640 (99.30)	*S. aureus* USA300
20 MRA	336,473	105	1432	289,966	287,842 (99.27)	*S. aureus* JH1
^*^21 MRA	766,395	107	8330	681,855	674,333 (98.90)	*S. aureus* USA300
^*^23 MRA	583,685	104	9419	464,855	459,591 (98.87)	*S. aureus* USA300
31 MRA	657,978	100	3419	596,716	595,028 (99.72)	*S. aureus* COL
55 MRA	489,670	96	585	298,828	289,039 (96.72)	*S. aureus* JH1
74 MRA	478,942	107	1129	410,085	405,586 (98.90)	*S. aureus* USA300
256 MRA	443,073	108	71	411,322	400,666 (97.41)	*S. aureus* USA300
***IN VITRO***						
1 MRA	479,683	105	2	404,995	397,930 (98.26)	
3 MRA	532,094	110	1	481,800	466,891 (96.91)	
4 MRA	474,698	108	0	414,247	407,187 (98.30)	
6 MRA	454,227	111	1	414,825	402,384 (97.00)	
20 MRA	442,793	105	2	403,797	399,087 (98.83)	
^*^21 MRA	440,061	106	8	408,518	401,531 (98.29)	
^*^23 MRA	466,089	109	2	444,640	434,200 (97.65)	
31 MRA	465,569	107	1	392,500	386,082 (98.36)	
55 MRA	401,893	100	4	357,369	349,760 (97.87)	
74 MRA	514,879	110	4	460,010	453,521 (98.59)	
256 MRA	449,617	107	2	385,719	380,309 (98.60)	

* *Subjects 21 and 23 represent the same individual for which samples were collected at different time points*.

### Identification of staphylococcal sequence reads

We mapped the sequence reads from each subject to a database of known staphylococcal genomes (available at ftp://ftp.ncbi.nlm.nih.gov/genomes/Bacteria/) to determine whether we could identify gene sequences from the *Staphylococcus* genome that were expressed upon exposure to human blood. We found that the vast majority of the *ex vivo* sequenced reads mapped to known staphylococcal genomes (mean 98.78 ± 0.32%), with a similar proportion of the *in vitro* sequenced reads (98.06 ± 0.19%) also mapping to known staphylococcal genomes. That a similar percentage of *ex vivo* and *in vitro* sequenced reads mapped to staphylococcal genomes, indicates the enrichment process was highly specific for bacterial RNA. Four of the 10 subjects likely harbored MRSA USA300 type strains based on their high percentage of mapping reads, while the other six subjects harbored strains that mapped to more traditional HA-MRSA strain types. Both samples 21MRA and 23MRA mapped to USA300 strains, indicating that this strain type was identical over the 48 h between samplings in that individual subject.

### *Ex vivo* vs. *in vitro* MRSA gene expression profiles

**Figure 1:**
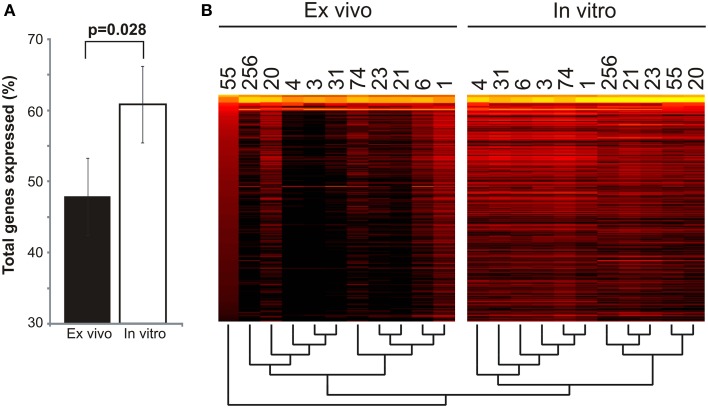
**Global gene expression of *Staphylococcus aureus* cultured in blood from human subjects (*ex vivo*) and *in vitro*. (A)**—mean percentage (± standard error) of total genes expressed *ex vivo* and *in vitro*. **(B)** —heatmap of gene expression profiles *ex vivo* and *in vitro* for all subjects. Each column represents an individual subject, and each row represent each individual *S. aureus* gene. The most highly expressed genes are shown in yellow, while genes that are not expressed (RPKM value of 0) are shown in black. A dendrogram representing the hierarchical clustering of each subject is shown below the heatmap.

**Figure 2:**
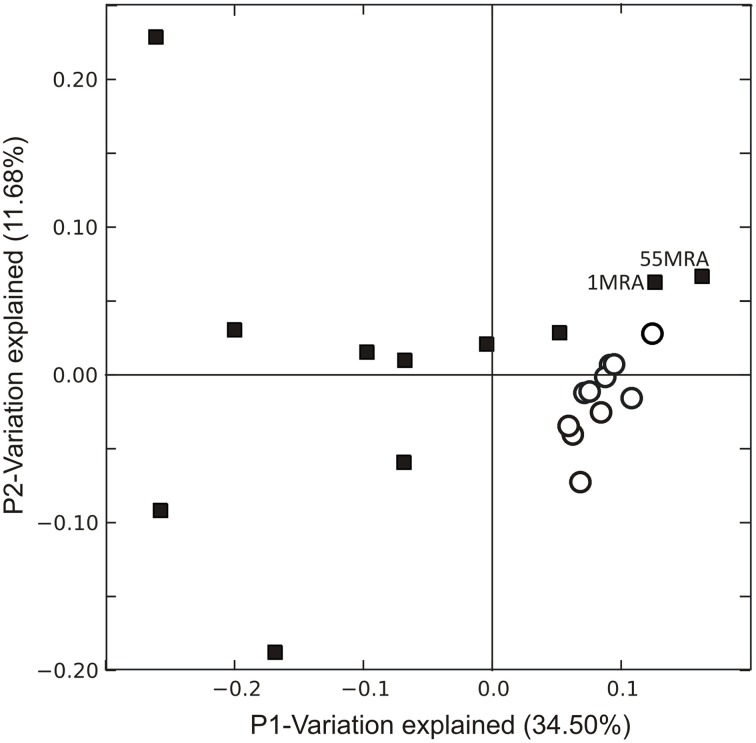
**Principal coordinates analysis of *Staphylococcus aureus* transcriptomes *ex vivo* (black squares) and *in vitro* (white circles)**. Beta diversity was determined using binary Sorensen distances based on RPKM values, and was used as input for the principal coordinates analysis.

We examined the overall gene expression from each subject both *ex vivo* and *in vitro* to determine whether there were global differences in gene expression that might signal adaptations to human blood. We found that there was a significant difference in the proportion of MRSA genes expressed, with far fewer genes expressed *ex vivo* (47.88 ± 5.39% *ex vivo* vs. 60.82 ± 1.07% *in vitro*; *p* = 0.028) (Figure 1A). As demonstrated by heatmap, many subjects had relatively limited global gene expression profiles *ex vivo* when compared to their *in vitro* counterparts (Figure 1B). Samples 21MRA and 23MRA from the same subject demonstrated similar but not identical patterns of gene expression. Overall, MRSA strains had patterns of gene expression that were reflective of either their *ex vivo* or *in vitro* environment (Figure 1B), suggesting that the limited patterns of gene expression *ex vivo* directly reflected adaptations to human blood. These data also were supported by principal coordinates analysis, which showed that much less heterogeneity amongst the *in vitro*-derived specimens than was observed for the *ex vivo* specimens (Figure 2).

### Subsystem specific gene expression in MRSA

**Figure 3:**
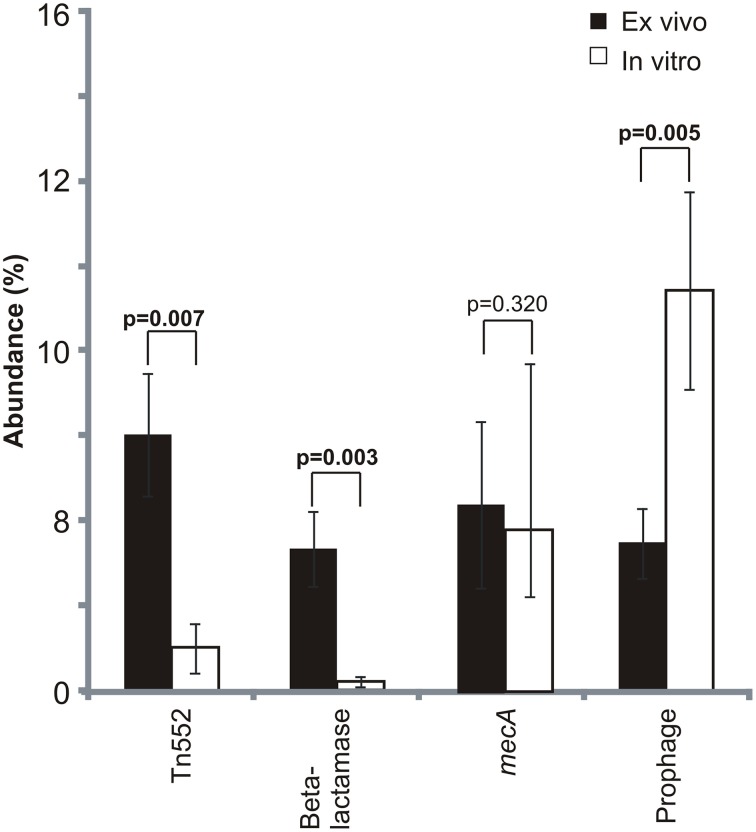
**Mean percentage (± standard deviation) of mobile element gene expression devoted to specific elements**. Mobile genetic element relative abundances were determined based on the proportion of each selected mobile element to the total mobile genetic element RPKM values. Mobile genetic elements were determined based on homologies to gene categories in the SEED database. *p*-values are shown above each mobile element. *Ex vivo* gene expression is represented by black bars and *in vitro* is represented by white bars.

We compared MRSA subsystem gene expression profiles to determine whether there were specific differences attributable to culture in media with human blood. There were significant differences (*p* = 0.05) in *ex vivo* and *in vitro* gene expression in numerous subsystems, including virulence/disease/defense, and mobile genetic elements among other subsystems identified (Supplemental Figure 2). In 9 of the 10 subjects studied, there was higher *ex vivo* expression of mobile elements than *in vitro* (Supplemental Figure 3), and significantly greater expression of prophage genes *in vitro* than *ex vivo* (Figure 3). Expression of beta lactamases involved in resistance to beta lactam antibiotics also was significantly increased on transposons (Tn552; *p* = 0.007) and on plasmids (*p* = 0.003). We found no significant differences in expression of the *mecA* penicillin binding protein gene, which also encodes resistance to beta lactam antibiotics.

We also examined the expression of virulence subsystems in MRSA *ex vivo* and *in vitro* to determine the impact of culture in media with human blood on MRSA virulence gene expression. We found that in seven of the 10 subjects studied, virulence gene expression was higher *in vitro* than *ex vivo* (Supplemental Figure 4), which suggests that human blood may have inhibitory effects on the expression of certain virulence genes. Despite the generally lower expression of virulence genes, some were more highly expressed *ex vivo*, including *Staphylococcus aureus* pathogenicity islands (SAPIs) and cytolysins (Supplemental Figure 5A). SAPIs are highly diverse phage-related chromosomal islands that insert into the genome at distinct sites (Novick and Subedi, 2007), while cytolysins generally are involved in the lysis of neutrophils (Queck et al., 2009). Only five of the MRSA isolates encoded PVL (lukS/F-PV), which was more highly expressed *ex vivo*. although the difference was not statistically significant (*p* = 0.120) (Supplemental Figure 5B).

Because of the high expression of beta lactamases despite the absence of beta lactam therapy in most subjects, we focused on the potential response of MRSA to other antibiotics. Each subject had been treated with vancomycin (a glycopeptide antibiotic that disrupts the cell wall of MRSA through the prevention of cross-linking at terminal d-alanine moieties). We found no significant difference in the expression of *ddl*, which encodes d-alanine moieties (Supplemental Figure 5B). *tcaA* also is involved in the response to glycopeptide antibiotics (Srinivasan et al., 2002), and was more highly expressed in all study subjects *ex vivo*, although the difference was not statistically significant (*p* = 0.101).

### Prophage expression inhibited by culture in media with blood

**Figure 4:**
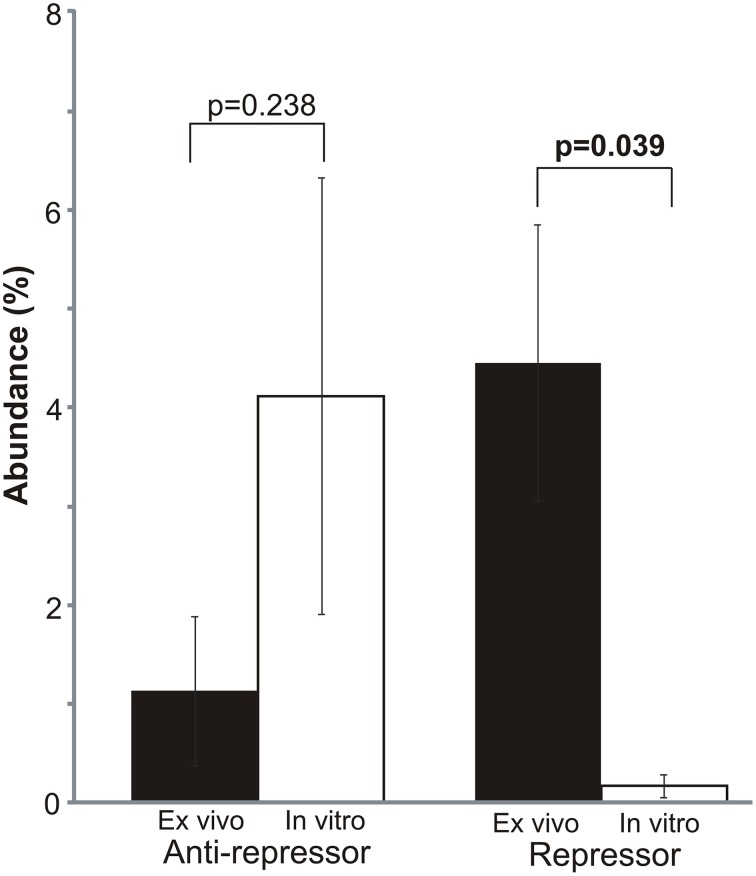
**Mean percentage (± standard deviation) of mobile element gene expression devoted to bacteriophage repressors and anti-repressors**. Repressor/antirepressor relative abundances were determined based on the proportion of each repressor/antirepressor to the total mobile genetic element RPKM values. Mobile genetic elements were determined based on homologies to gene categories in the SEED database. *p*-values are shown above each element. *Ex vivo* gene expression is represented by black bars and *in vitro* is represented by white bars.

Because of the significantly higher expression of mobile genetic elements *ex vivo* (Supplemental Figure 3), and the greater expression of prophage genes *in vitro* (Figure 3), we investigated whether there may be specific differences identified in bacteriophage gene expression *in vitro* and *ex vivo*. There were significant differences in the expression of phage repressors, which were significantly more highly expressed *ex vivo* (*p* = 0.039) (Figure 4). Similarly, we also found high expression of phage anti-repressors *in vitro*, although those differences were not statistically significant (*p* = 0.238). The higher expression of repressors and antirepressors *ex vivo* and *in vitro*, respectively, suggests that there were specific interactions with human blood that resulted in inhibition of prophage progression to lytic gene expression.

**Figure 5:**
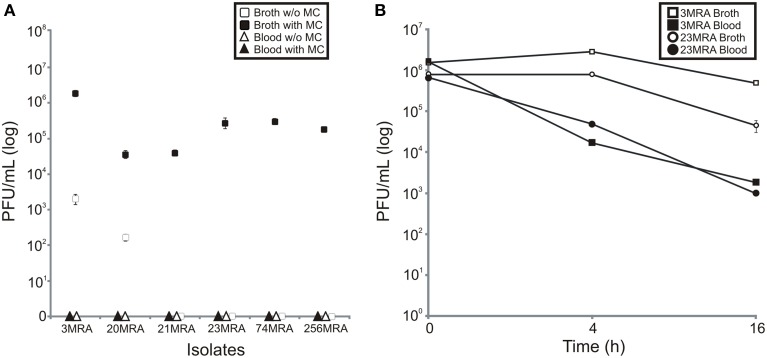
**Production of phage from MRSA strains in the presence of human blood, or BHI broth with and without mitomycin C (A), and inhibition of infectivity of phage produced in human blood or BHI broth (B)**. Mean plaque forming units (log) (± standard deviation) from three separate experiments is demonstrated. In **(A)**, BHI broth with and without mitomycin C are represented by black and white squares, respectively, and blood with and without mitomycin C are represented by black and white triangles, respectively. In **(B)**, phage 3MRA in the presence of BHI broth and blood is represented by white and black squares, respectively, and phage 23MRA in the presence of BHI broth and blood is represented by white and black circles, respectively. Standard deviation bars are demonstrated on each panel.

To test whether culture in media with human blood was inhibitory to prophage expression, we stimulated prophage using mitomycin C in each of the 11 isolated MRSA strains. We found that 6 of the 11 strains produced viable phage in Brain Heart Infusion broth (BHI) in the presence of mitomycin C (including strains 3MRA, 20MRA, 21MRA, 23MRA, 74MRA, and 256MRA); however, the production of phage was inhibited in all six strains by the presence of human blood in culture media (Figure 5A). Interestingly, there was spontaneous induction of phage in strains 3MRA and 20MRA, but the number of phage were two to three logs lower than were produced in the presence of mitomycin C. We also tested whether the reduction in phage identified in the presence of blood may be related to inhibition of phage infectivity rather than a decrease in phage production. We found that for both 3MRA and 23MRA there was a decrease in observed PFUs by 1.5 to 2 logs, indicating that infectivity was diminished in the presence of blood (Figure 5B). Because phage adsorption has previously been shown to be inhibited by immunoglobulins (Martin and White, 1968; Nordstrom et al., 1974), it is likely that diminished adsorption was responsible for the decrease in infectivity. The fact that infectivity was only partially inhibited (Figure 5B) suggests the lack of PFUs produced in culture with blood (Figure 5A) cannot be completely explained by inhibition of adsorption. These data are highly suggestive that expression and adsorption of these phage are inhibited by life in the human bloodstream.

### Identification of inhibited prophage

**Figure 6:**
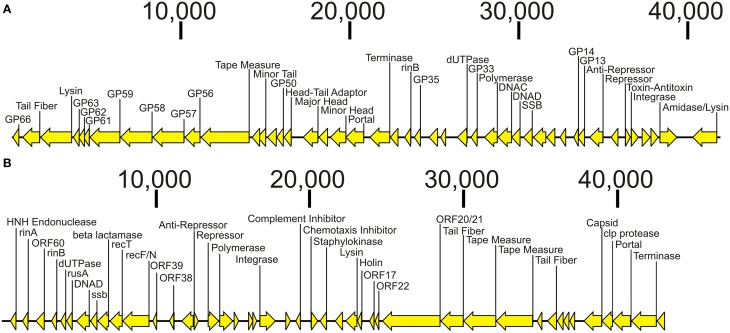
**Diagram of bacteriophage genomes sequenced from the virions from Subjects 3MRA (A) and 23MRA (B)**. Putative ORFs and their direction are indicated by the arrows. ORFs that had significant homologs (BLASTP E-score <10^−5^) are indicated by the text above each arrow. In **(A)**, those ORFs that had synteny with *Staphylococcus* phage PhiETA2 are labeled GP66, GP63, GP62, GP61, GP59, GP58, GP57, GP56, GP50, GP35, GP33, GP14, and GP13. In **(B)**, those ORFs that had synteny with *Staphylococcus* phage PhiPVL are labeled ORF60, ORF39, ORF38, ORF22, ORF21, ORF22, and ORF17. Putative functions for each ORF are labeled when available. The length of each contig is denoted at the top of each panel.

**Figure 7:**
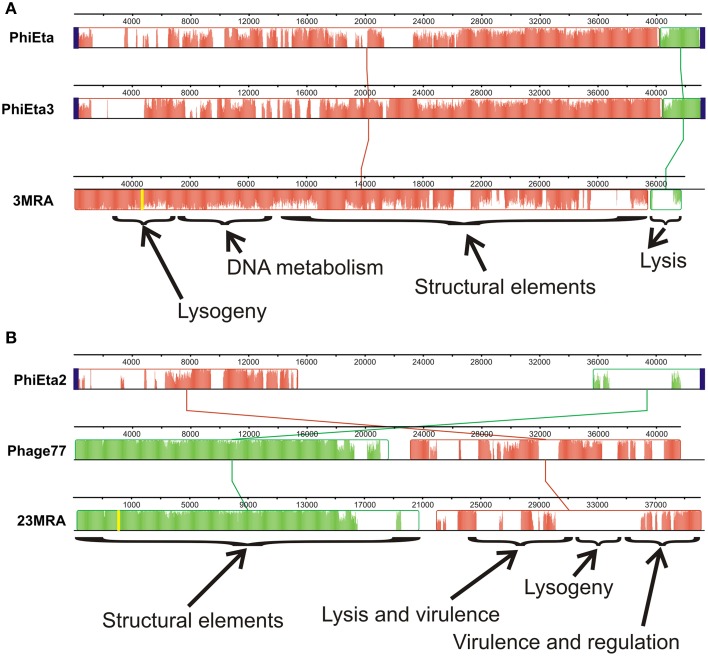
**Alignments of phage 3MRA (A) and phage 23MRA (B) with various other known staphylococcal phage**. The boxes represent segments of each phage that are relatively well conserved amongst the aligned phage and the lines between the boxes represent the relative position of conserved segments of each phage in the different genomes. Diagonal lines represent potential genome rearrangements. Relative locations in the genomes of each phage are demonstrated by the nucleotide numbers above each phage. The yellow lines represent the sites of the first nucleotide in the assembled 3MRA and 23MRA phage contigs. Some phage segments shown by the arrows contain conserved genes representing structural elements, DNA metabolism, lysis, lysogeny, virulence, and regulation. The height of the colors across each box represents the average conservation in that phage segment across the phage examined. *attP* sites, which represent the sites on phage genomes where they integrate into their host genomes, are represented by blue boxes. *attP* sequences were not determined for phage 3MRA and 23MRA.

We sequenced the phage from subjects 3MRA and 23MRA to determine which specific phage were inhibited in culture media with blood. We isolated phage 3MRA and 23MRA starting from single plaques, then purified and sequenced the DNA directly from the virions. We produced a total of 241,857 reads for phage 3MRA, of which 234,536 (97%) assembled into a single 42,141bp contig with 1177X average coverage (Figure 6A). We also sequenced 216,080 reads for phage 23MRA, of which 197,211 (91%) assembled into a single 43,114bp contig with 961X average coverage (Figure 6B). Based on BLASTN analysis, there were no nearly identical matches for phage 3MRA, but it was similar and shared synteny with *S. aureus* siphoviruses phiETA and phiETA3 (Figure 7A). Phage 23MRA was virtually identical to a prophage found in the genome of *S. aureus* USA300 TCH1516, which also is closely related to *S. aureus* siphoviruses phage 77, and phiETA2 (Figure 7B). Genome alignments shows that phage 23MRA has much greater similarity to Phage 77 than to phiETA2. Each phage sequenced had homologs to structural genes (head, tail, and portal), replication machinery (polymerases), integration genes (integrases), lysis and packaging machinery (lysins and terminases), genes that control transcription (repressors/anti-repressors), and virulence genes (toxin-antitoxin, complement inhibitor, chemotaxis inhibitor, and staphylokinase). All ORFs identified in phage 3MRA and 23MRA phage had homologs to staphylococcal phage genes or genes previously identified in staphylococcal genomes. These data specifically identify prophage whose expression is inhibited by culture in media with blood, and the profound similarity between these phage and many previously identified staphylococcal prophage suggests that they may also be inhibited by human blood.

## Discussion

*Staphylococcus aureus* is highly prevalent and responsible for substantial morbidity and mortality. Its epidemiology has shifted, resulting in methicillin-resistant strains having similar prevalence to methicillin-sensitive strains in clinical settings. Because studies generally identify single MRSA genotypes from sterile site infections (Young et al., 2004), most MRSA infections are believed to be caused by single rather than multiple different genotypes. There was no evidence based on the MRSA culture data and susceptibility patterns of mixed infections in the 10 subjects studied. While much is known about its pathogenicity, the *in vivo* gene expression of MRSA and its lysogenic phage have yet to be thoroughly examined in humans. Our technique for characterizing MRSA *ex vivo* gene expression was unique in that it involved examining the gene expression of MRSA cultured directly from the blood of different human subjects with bloodstream infections without any separate *in vitro* culture steps, followed by comparisons with *in vitro* growth to identify differences that might be attributable to life in the human bloodstream. While not technically an *in vivo* technique because of the separate culture step, we identified numerous differences between *ex vivo* and *in vitro* MRSA gene expression profiles, with the lysogenic phage providing the most notable differences. Although the group of subjects we studied was relatively heterogeneous in their underlying comorbidities, each had invasive MRSA bloodstream infections. The heterogeneity in their comorbidities and individualized medical therapies may be responsible for the variability observed in gene expression from each subject (Figure 1). Because it took approximately 72 h to identify those subjects who had MRSA bloodstream infections, most of the subjects enrolled in this study had either cleared their bacteremia or were deceased by the time a diagnosis was made. This severely limited our ability to obtain further specimens to test biological replicates. Because some of the subjects were critically ill and treated with complex antibiotic combinations (Table 1), the features of their immune responses and antibiotic regimens would be difficult to replicate *in vitro*, which further adds to the uniqueness of this cohort and the responses of the MRSA isolates studied. The differences in MRSA gene expression profiles observed cannot be solely attributed to antibiotics, as there were different gene expression profiles observed in subjects treated with the same antibiotic regimens.

We were highly interested in the expression of mobile genetic elements (including phage, plasmids, and transposons) associated with bacterial infections in the human bloodstream, as they may be the primary means by which new gene functions are transmitted in nature. Beta lactamases expressed on mobile elements *ex vivo* were significantly overexpressed compared to *in vitro* (Figure 3), suggesting that their expression may be part of the organism's stress response to human blood. Because most of the subjects studied were not treated with beta lactam antibiotics, the regulation of these beta lactamases likely is independent of beta lactam therapy. While beta lactamases were more highly expressed *ex vivo*, *mecA* expression was stable under each growth condition. Because the MecA penicillin binding protein is involved in the normal processing of the MRSA cell wall, its expression might not be expected to be substantially altered when cultured in media with human blood.

While there has been some prior analysis of the effect of human blood on prophage (Gaidelyte et al., 2007), our *ex vivo* data strongly suggest that repression of certain prophage is characteristic of MRSA invasive bloodstream infections. This was supported by the higher *ex vivo* expression of repressor genes (Figure 4), which are essential for the inhibition of the phage lytic genes. An inverse effect was noted *in vitro*, where the higher expression of anti-repressor genes was indicative of the expression of the phage lytic module. We were unable to place the MRSA genomes into lineage groups (Lindsay et al., 2006; McCarthy and Lindsay, 2010) based on the presence/absence of mobile genetic elements because the heterogeneity in gene expression and the potential for prior horizontal gene transfers (McCarthy et al., 2012a,b) could have led to erroneous results. There also was altered expression of site-specific recombinases in this cohort, but their potential dual roles in integration and excision complicates their interpretation (Hanssen and Ericson Sollid, 2006; Malachowa and Deleo, 2010).

In addition to altered phage expression in response to blood in culture media, we found that infectivity of the phage also was diminished, likely through inhibition of adsorption (Figure 5) (Martin and White, 1968; Nordstrom et al., 1974). These results suggest that the transport and adsorption of phage particles, as well as the transmission of phage-encoded genes, may be restricted during MRSA bloodstream infections in humans. We also identified certain phage that were specifically inhibited by culture in media with human blood (Figure 6), and their strong similarities to prophage found in other MRSA isolates (Figure 7) suggests that they also may be inhibited by blood. Not all MRSA isolates had produced phage particles that we could detect using acceptor strain RN10950. We also utilized strain RN4220 (a restriction deficient *S. aureus* strain cured of three prophage) (Novick, 1967), but were unable to detect phage from the other MRSA isolates. Our study differs significantly from a prior study documenting the effects of human blood on staphylococcal phage. In that study, the authors concentrated specifically on phage that were expressed during culture in media with human blood (Gaidelyte et al., 2007), whereas our study identifies those phage that were inhibited by culture with human blood. Also, in contrast to that study, our data on these specific MRSA phage shows that infectivity was diminished in the presence of blood (Figure 5).

The production of viable phage from prophage in the MRSA genomes in this study directly supported the RNA sequencing data. We observed a substantial over-expression of phage repressors *ex vivo* (Figure 4), which correlated directly with the inhibition of phage production in human blood (Figure 5). Although most MRSA isolates in our study carried integrases, which often indicate the presence of prophage, not all strains produced viable prophage that we could detect with our acceptor strains. This suggests that some of our MRSA strains carried prophage remnants and/or cryptic prophage rather than viable prophage (Canchaya et al., 2003; Casjens, 2003). With the exception of isolate 6MRA, most CA-MRSA strains produced viable phage, compared to only one HA-MRSA strain (3MRA). The phage we identified (Figure 6) had virulence genes such as staphylokinase (involved in tissue destruction), toxin/antitoxin systems (may be involved in defense, drug tolerance, programmed cell death, and growth control) (Nolle et al., 2013), and genes for an immune escape complex (involved in immune evasion) (Goerke et al., 2006), which suggests that these phage may play several different roles in MRSA pathogenesis.

## Conclusions

While some studies have profiled *S. aureus in vitro* behavior (Renzoni et al., 2006; Sass et al., 2008) and gene expression profiles in mouse models (Allard et al., 2006; Chaffin et al., 2012), the gene expression of their bacteriophage and other mobile genetic elements have not been examined *in vivo*. While our *ex vivo* model includes a separate culture step, the blood culturing step was absolutely necessary to first identify those human subjects that had invasive bloodstream infections. The benefit of the model was that we could isolate each strain *in vitro*, and thus discern differences in bacteriophage and mobile genetic elements gene expression profiles between *ex vivo* and *in vitro* that may have represented adaptation to life in the human bloodstream. The expression of mobile genetic elements was amongst the most significant differences between gene expression profiles *ex vivo* and *in vitro*, and these differences identified may have consequences for the human host. From the over-expression of beta lactamases on plasmids and transposons *ex vivo*, to the repression of prophage expression and adsorption during culture in media with human blood, differences in the expression of mobile genetic elements were characteristic of the MRSA *ex vivo* response. As we continue to study the behavior of human pathogens, *ex vivo* studies such as this provide further insights into the gene expression patterns in pathogens and their viruses as they adapt to life in the human host.

## Materials and methods

### Ethics statement

Human subject involvement in this study was approved by the University of California, San Diego Administrative Panel on Human Subjects in Medical Research. The study was certified as category 4 exempt, which includes research involving the collection or study of existing data, documents, records, pathological specimens, or diagnostic specimens, if the information is recorded in such a manner that subjects cannot be identified, directly or through identifiers linked to the subjects.

### Human subjects and culture conditions

We sampled blood from 10 human subjects with invasive bloodstream infections (Table 1). Samples 21MRA and 23MRA were taken from the same subject approximately 48 h apart for a total of 11 different samples from 10 subjects. From each subject, 8–10 mL of blood was inoculated directly at bedside into 30 ml of media in BD Bactec Plus aerobic culture vials (BD Diagnostic Systems, Sparks, MD) and incubated for approximately 4–8 h until positive by fluorescent detection using the Bactec FX system (Zadroga et al., 2013). Positive cultures were gram stained, grown on sheep blood agar plates, and subjected to a coagulase test for a presumptive identification of *Staphylococcus aureus*. Identification and susceptibility testing was performed using the BD Phoenix system (Stefaniuk et al., 2003) to confirm the presence of MRSA in the bloodstream of each subject. Susceptibilities were based on MIC (minimum inhibitory concentration) breakpoints using Clinical and Laboratory Standards Institute guidelines (CLSI, 2012). Each isolate was assayed for susceptibility to antibiotics cefazolin, clindamycin, daptomycin, linezolid, oxacillin, rifampin, trimethoprim/sulfamethoxazole, and vancomycin. Clindamycin susceptibility was verified by D-test (Woods, 2009). A minimum of 3 mL of each positive sample in the standard aerobic culture vial was immediately processed in the *ex vivo* arm of this study. For *the in vitro* growth conditions, each identified MRSA strain was grown on blood agar plates, and reconstituted in tryptic soy broth at an OD600 of 0.1. One milliliter of this suspension was diluted in 7 ml of sterile normal saline and inoculated into the BD Bactec Plus aerobic culture vials and incubated for approximately 3–6 h until positive by fluorescent detection using the Bactec FX system. One milliliter of this suspension was immediately processed in the *in vitro* arm of this study.

### MRSA growth conditions

OD_600_ values for each isolate grown in the BD Bactec Plus aerobic vials was determined at the time of fluorescence detection (approximately 3.5 to 4.5 h, depending on the isolate). To determine the growth phase at the time of fluorescence detection, MRSA isolates were grown for 16 h in BD Bactec Plus aerobic broth and reconstituted to an OD_600_ of 0.1. Cultures were then incubated at 35°C in BD Bactec Plus aerobic broth with gentle agitation and OD_600_ values for each isolate was determined at 15 min intervals over a 24 h period to construct growth curves. CFU/mL for each isolate under *in vitro* conditions were determined by plating 100 μL of serial dilutions onto BHI agar plates and incubated at 35°C for 16 h. Because the presence of the blood prohibited us from using direct comparisons of OD_600_ values between the *ex vivo* and *in vitro* cultures, we determined the CFU/mL counts from each subject *ex vivo* to estimate the growth phase at the time of fluorescence detection by plating 100 μL of serial dilutions on BHI agar plates. All *ex vivo* and *in vitro* cultures were found to be in early to mid log phase at the time of detection. Two volumes of RNA protect (Qiagen, Valencia, CA) was added directly to the cultures, and they were pelleted and stored at −20°C until RNA extraction.

### RNA extraction, enrichment, and sequencing

RNA from both the *ex vivo* and *in vitro* samples were processed identically from all subjects and MRSA isolates. Total RNA was extracted using the Mirvana kit (Life Technologies, Grand Island, NY), with the inclusion of a bead-beating step for 20 min with Lysing-Matrix B (MP Bio, Santa Ana, CA). Total RNA then was enriched for microbial RNA using MicrobEnrich (Life Technologies), and further enriched for mRNA using MicrobExpress (Life Technologies) and MegaClear (Life Technologies), which are designed to remove ribosomal RNAs. Enriched RNA then was prepared for sequencing through the construction of cDNA libraries using the Ion Total RNA-Seq kit (Life Technologies), and subjected to successive rounds of Ampure bead purification (Beckman-Coulter, Brea, CA) to remove small cDNAs. Libraries were quantified using an Agilent Bioanalyzer HS DNA Kit (Agilent, Santa Clara, CA) and then were sequenced on a 314 chips using an Ion Torrent Personal Genome Machine (Rothberg et al., 2011), producing an average of 559,129 reads per subject of mean length 106 nucleotides. All sequence data produced in this study are available in the MG-Rast database (metagenomics.anl.gov/) under the project name “MRSA_RNAseq_Study” or project #2278.

### Processing of RNA sequences

Sequencing reads were trimmed according to modified Phred quality scores of 0.5 using CLC Genomics Workbench 4.65 (CLC bio USA, Cambridge, MA). The remaining reads were further processed for quality control by removing reads with substantial length variation (reads <50 nucleotides or >200 nucleotides), or reads where ≥25% of the length was due to homopolymers tracts. Each transcriptome was screened for contaminating human nucleic acids using BLASTN analysis (E-score <10^−5^) against the human reference database available at ftp://ftp.ncbi.nlm.nih.gov/genomes/H_sapiens/. Any reads homologous to human sequences were removed prior to further analysis. Both *in vitro* and *ex vivo* reads from each subject were mapped to a database of staphylococcal genomes (available at ftp://ftp.ncbi.nlm.nih.gov/genomes/Bacteria/) to determine the percentage of reads that mapped to *Staphylococcus* and to which individual strains they mapped best. Reads from each subject mapped best to known MRSA genomes in all subjects under each growth condition (Table 2).

### Analysis of transcriptomes

Each *ex vivo* and *in vitro* transcriptome were mapped against USA300 MRSA strain FPR3757 using CLC Genomics Workbench 4.65. RPKM (reads per kilobase per million) values were determined based on the read mappings, and values were normalized for each subject and growth condition. A heatmap demonstrating the distribution of gene expression based on normalized RPKM values was generated using CLC Genomics Workbench 4.65. Virtually identical heatmaps also were generated when reads were mapped against genomes of other MRSA strains including COL, JH1, Mu50, TCH1516, and Newman. RPKM values also were used as input for principal coordinates analysis, and were performed based on binary Sorensen distances using Qiime (Caporaso et al., 2010).

Analysis of gene expression from different subsystems in MRSA were determined by blastX analysis of the SEED database using MG-Rast (E-score <10^−5^) (Meyer et al., 2008). Mobile genetic element and virulence gene expression was determined based on the proportion of each selected mobile element or virulence gene to the total mobile genetic element or virulence gene RPKM values. Subsystems such as mobile genetic elements and virulence genes were determined based on homologies to genes designated to those functions in the SEED database (Meyer et al., 2008). Statistical significance was determined by comparing the means for all subjects for all subsystems between the *ex vivo* and *in vitro* subject groups by two-tailed *t*-tests using Microsoft Excel 2007 (Microsoft Corp., Redman, WA). Analysis of the expression differences between *in vitro* and *ex vivo* groups for individual genes also were determined by comparisons of means. The data for each individual gene were compared between MG-Rast and the normalized RPKM values obtained from CLC Genomics Workbench 4.65 to verify that they produced similar results.

### Prophage stimulation and sequencing

Each MRSA strain was grown in BHI broth (Becton, Dickinson, MD, USA) for approximately 3 h at 37°C with shaking at 200 rpm to an OD_600_ of 0.5. The average number of cells was similar between each MRSA strain (1.84 ± 0.45 × 10^8^ Colony Forming Units (CFU)/mL). Each strain then was centrifuged for 10 min at 10,000 rpm, pellets re-suspended in 500 μL of BHI broth or human blood (Novick, 1963), and mitomycin C added to a final concentration of 2 μg/mL. The human blood used was drawn into heparin tubes (BD Diagnostic Systems, Sparks, MD) from healthy donors. MRSA cultures without the addition of mitomycin C were used as experimental controls and were without evidence of any prophage production, with the exception of isolates 3MRA and 20MRA. Each was left for 16 h at 32°C with shaking at 50 rpm. To determine the number of phage produced, cultures were centrifuged for 5 min at 14, 000 rpm to remove cellular debris, and filtered through 0.2 μm pore membrane filters (25 mm, Whatman GE Healthcare Life Sciences). Supernatants were cultured for the presence of any residual bacteria, and no bacteria could be cultured from any. We tested the supernatants for the presence of viable phage using the double layer method (Novick, 1991). Briefly, *S. aureus* strain RN10950 (*S. aureus* Newman strain with all four prophage deleted) (Bae et al., 2006) was grown for 4 h at 37°C with shaking at 200 rpm to an OD_600_ of 1.0. Then, 100 μL of *S. aureus* RN10950 and 100 μL of the phage supernatants were added to 3 mL of phage top agar and poured onto phage bottom agar plates (Novick, 1991). Plates were incubated at 32°C for 16 h and viral plaques were enumerated and reported as Plaque Forming Units (PFU)/mL.

To determine if blood may inhibit phage infectivity, phage 3MRA and 23MRA were added to 1 mL of BHI or fresh human blood and incubated at 32°C with shaking at 50 rpm to maintain the conditions used in induction experiments. Aliquots were collected at 0, 4, and 16 h to determine the infectivity of phage. Blood cells were removed prior to plating phage by centrifugation at 14,000 rpm for 5 min and filtered through 0.45 μm filters. The phage in BHI also was centrifuged and filtered for consistency. *S. aureus* strain RN10950 was grown for 4 h at 37°C with shaking at 200 rpm to an OD600 of 1.0. Then, 100 μL of RN10950 and 100 μL of BHI or blood containing the seeded phage were serially diluted and added to 3 mL of phage top agar and poured onto phage bottom agar plates. Plates were incubated at 32°C for 16 h and viral plaques were enumerated and reported as PFU/mL. All experiments were performed in triplicate.

Phage were isolated as previously described (Santiago-Rodriguez et al., 2010). Briefly, viral plaques were retrieved using a sterile pipette and placed in 500 μL of 1× PBS. The plug was dislodged using a sterile pipette and centrifuged at 14,000 rpm for 5 min. The supernatant was collected and further propagated by adding 100 μL of the phage supernatant and 100 μL (OD_600_ of 1.0) of *S. aureus* RN10950 to 3 mL of top agar. The mixture was poured onto phage bottom agar plates and incubated at 32°C for 24 h. The top agar was collected and centrifuged at 7500 rpm for 15 min. The supernatant was collected and filtered sequentially through 0.45 μm filters and 0.22 μm filters. The propagation step was repeated until complete lysis was observed. Supernatants then were purified on a cesium chloride gradient according to previously described protocols for isolation of viruses (Pride et al., 2012). Only the fraction with a density corresponding to most known bacteriophage (Murphy et al., 1995) was retained, further purified on Amicon YM-100 protein purification columns (Millipore, Inc., Bellerica, MA), treated with DNase I, and subjected to lysis and DNA purification using the Qiagen UltraSens virus kit (Qiagen, Valencia, CA). Resulting DNA was fragmented to roughly 200 to 400 bp using a Bioruptor (Diagenode, Denville, NJ), and utilized as input to create libraries using the Ion Plus Fragment Library Kit according to manufacturer's instructions. Libraries then were sequenced using a 314 chip on an Ion Torrent Personal Genome Machine (PGM; Life Technologies, Grand Island, NY) (Rothberg et al., 2011) producing an average read length of approximately 216 bp for each sample. Sequence reads were trimmed according to modified Phred scores of 0.5 using CLC Genomics Workbench 4.65 (CLC bio USA, Cambridge, MA). Any low complexity reads (where >25% of the length were due to homopolymer tracts), reads with substantial length variation (<50 nucleotides or >300 nucleotides), or reads with ambiguous characters also were removed prior to further analysis. Remaining reads were assembled using CLC Genomics Workbench 4.65 based on 98% identity with a minimum of 50% read overlap, which are more stringent than criteria developed to discriminate between highly related viruses (Breitbart et al., 2002). The consensus sequence for each assembled phage was constructed according to majority rule. Viral contigs were analyzed using FGenesV (Softberry Inc, Mount Kisco, NY) for ORF prediction, and individual ORFs analyzed using BLASTP analysis against the NCBI non-redundant database (Escore <10^−5^). Alignments of phage genomes were performed with progressiveMauve using the default settings (Darling et al., 2010). Sequences of each *Staphylococcus* phage are available in Genbank under accession numbers KJ452291 and KJ452292. The reverse complements of these phage genome sequences were utilized in the genome alignments.

## Author contributions

Conceived and designed experiments: DTP and MBJ. Performed the experiments TSR, MN, and ML. Analyzed the data: DTP, TSR, and MBJ. Wrote the manuscript: DTP and TSR. All authors have read and approved this manuscript.

### Conflict of interest statement

The authors declare that the research was conducted in the absence of any commercial or financial relationships that could be construed as a potential conflict of interest.

## Supplementary material

Supplemental Figure 1 **Growth curves for MRSA isolates**. Each isolate is shown in separate panels with their respective OD_600_ measurements at 15 min intervals on the y-axis and the time over a period of 24 h period shown on the x-axis. The time of fluorescence detection under *in vitro* (blue circles) and *ex vivo* (red circles) conditions are shown for each subject in each panel.

Supplemental Figure 2 **Mean percentage (± standard deviation) of all gene expression from each subsystem in *Staphylococcus aureus* transcriptomes *ex vivo* and *in vitro***. The upper panel shows subsystems that were significantly different between *ex vivo* and *in vitro* samples, and the lower panel shows subsystems that were not significantly different. *p*-values are shown to the right of each subsystem. *Ex vivo* gene expression is represented by black bars and *in vitro* is represented by white bars.

Supplemental Figure 3 **Abundance (%) of mobile element gene expression in *Staphylococcus aureus* transcriptomes for all subjects**. Specimens 21MRA and 23MRA are from the same subject 48 h apart.

Supplemental Figure 4 **Abundance (%) of virulence gene expression in *Staphylococcus aureus* transcriptomes for all subjects**. *Ex vivo* gene expression is represented by black bars and *in vitro* is represented by white bars. Specimens 21MRA and 23MRA are from the same subject 48 h apart.

Supplemental Figure 5 **Mean percentage (± standard deviation) of the virulence subsystem devoted to individual elements**. *p*-values are shown above each virulence factor. *Ex vivo* gene expression is represented by black bars and *in vitro* is represented by white bars. **(A)** shows Staphylococcal Pathogenicity Islands (SaPIs) and cytolysins, and **(B)** shows *tcaA* (teicoplanin-resistance), *ddl* (involved in cell wall synthesis) and *lukS-PV* and *lukF-PV* (encoding Panton-Valentine leukocidin).
