# Supplementary figures and images for: Identification of staphylococcal phage with reduced transcription in human blood through transcriptome sequencing
Source: Front Microbiol. 2015 Mar 24;6:216. doi: 10.3389/fmicb.2015.00216 (PMC4447126; doi:10.3389/fmicb.2015.00216)

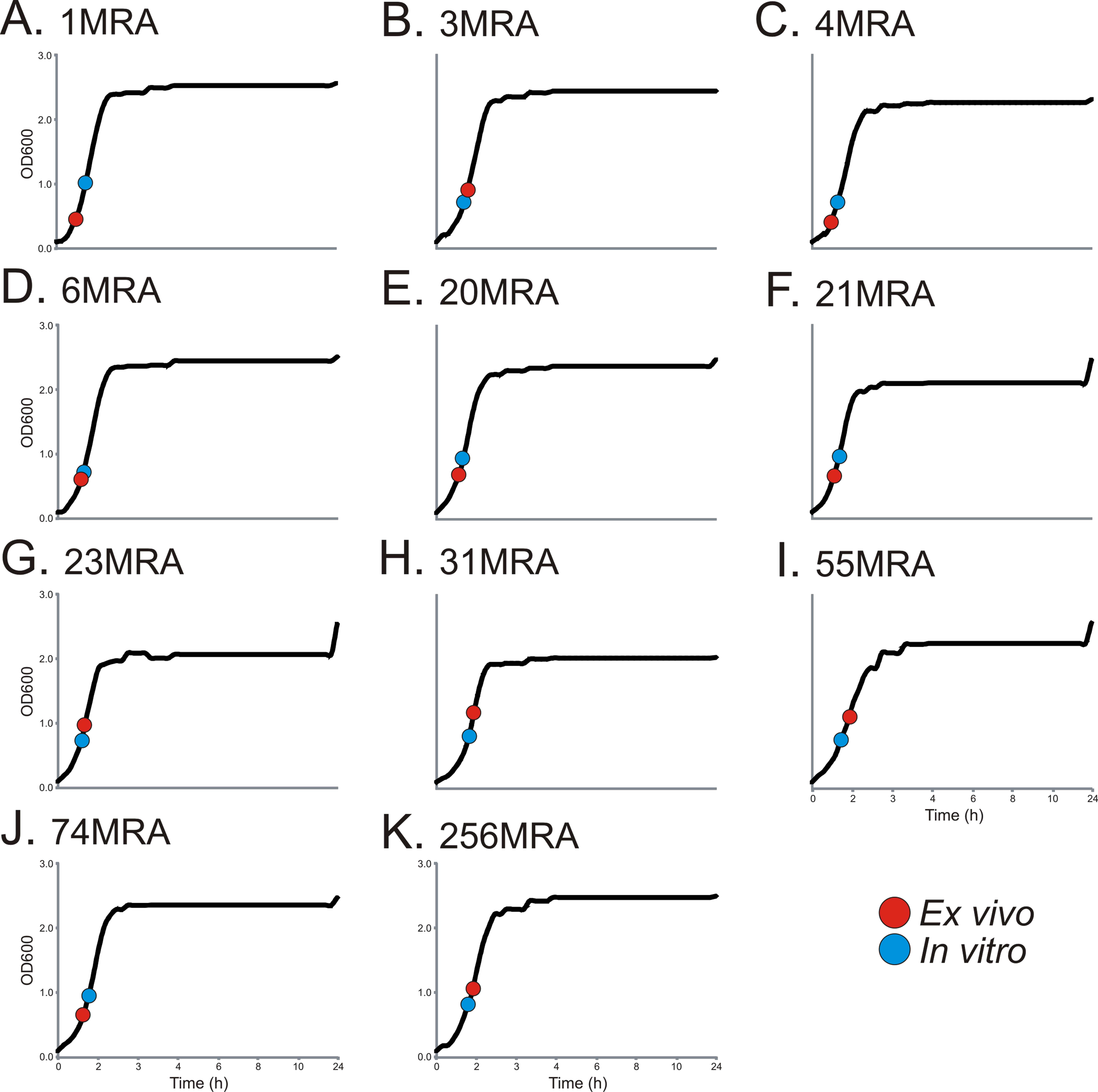

Supplement: Supplemental Figure 1 — Growth curves for MRSA isolates. Each isolate is shown in separate panels with their respective OD600 measurements at 15 min intervals on the y-axis and the time over a period of 24 h period shown on the x-axis. The time of fluorescence detection under in vitro (blue circles) and ex vivo (red circles) conditions are shown for each subject in each panel. [file Image1.TIF]

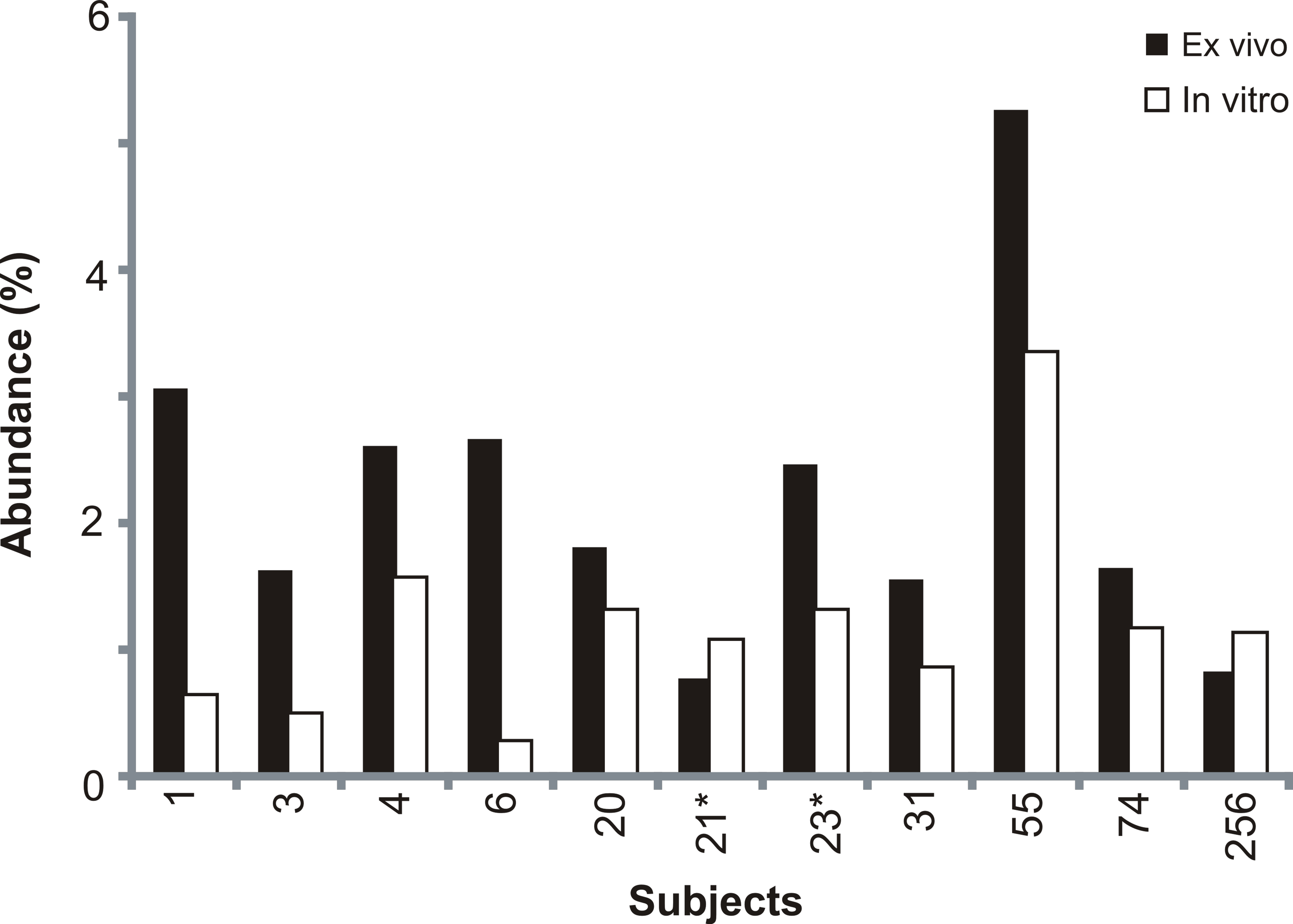

Supplement: Supplemental Figure 3 — Abundance (%) of mobile element gene expression in Staphylococcus aureus transcriptomes for all subjects. Specimens 21MRA and 23MRA are from the same subject 48 h apart. [file Image3.TIF]

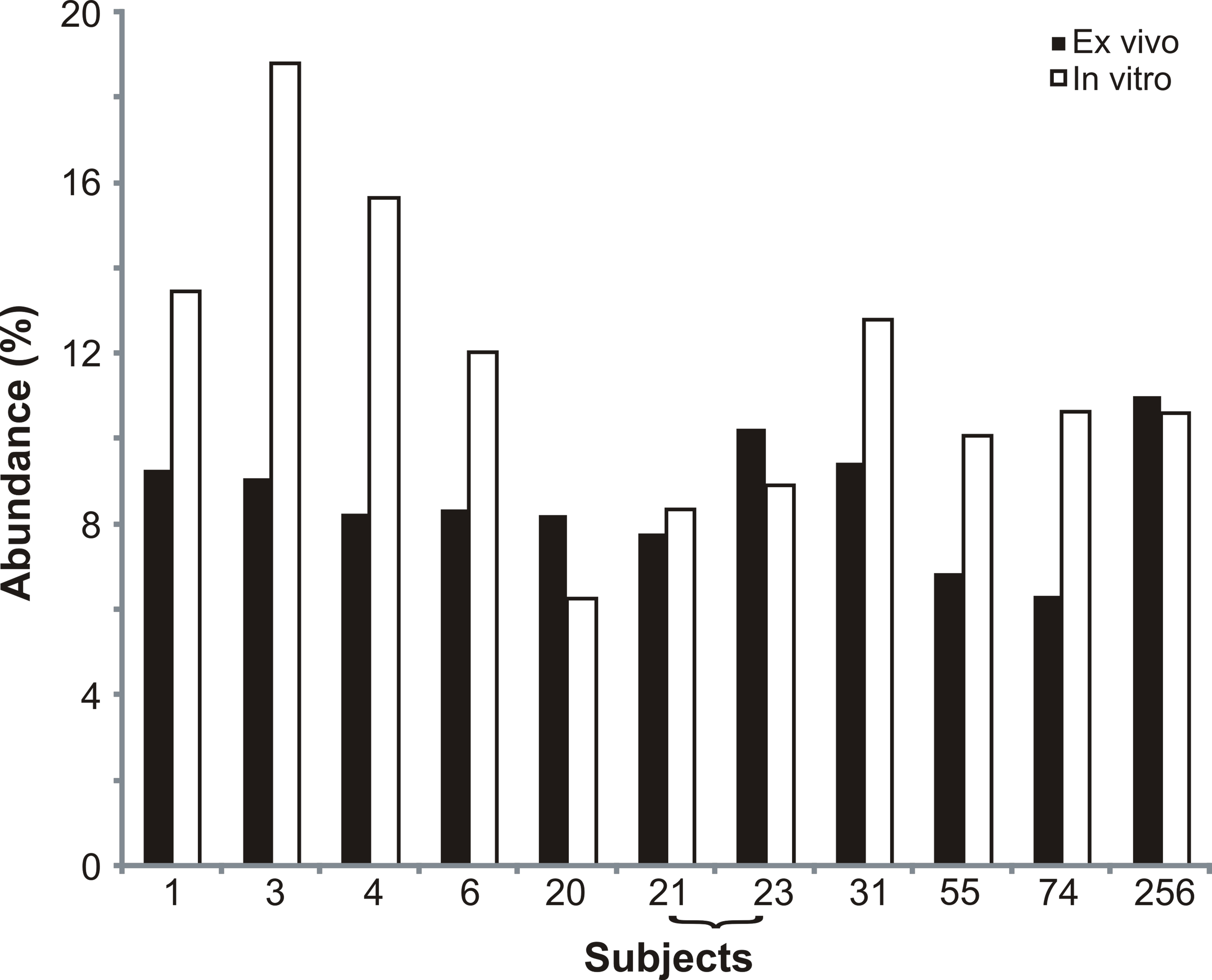

Supplement: Supplemental Figure 4 — Abundance (%) of virulence gene expression in Staphylococcus aureus transcriptomes for all subjects. Ex vivo gene expression is represented by black bars and in vitro is represented by white bars. Specimens 21MRA and 23MRA are from the same subject 48 h apart. [file Image4.TIF]

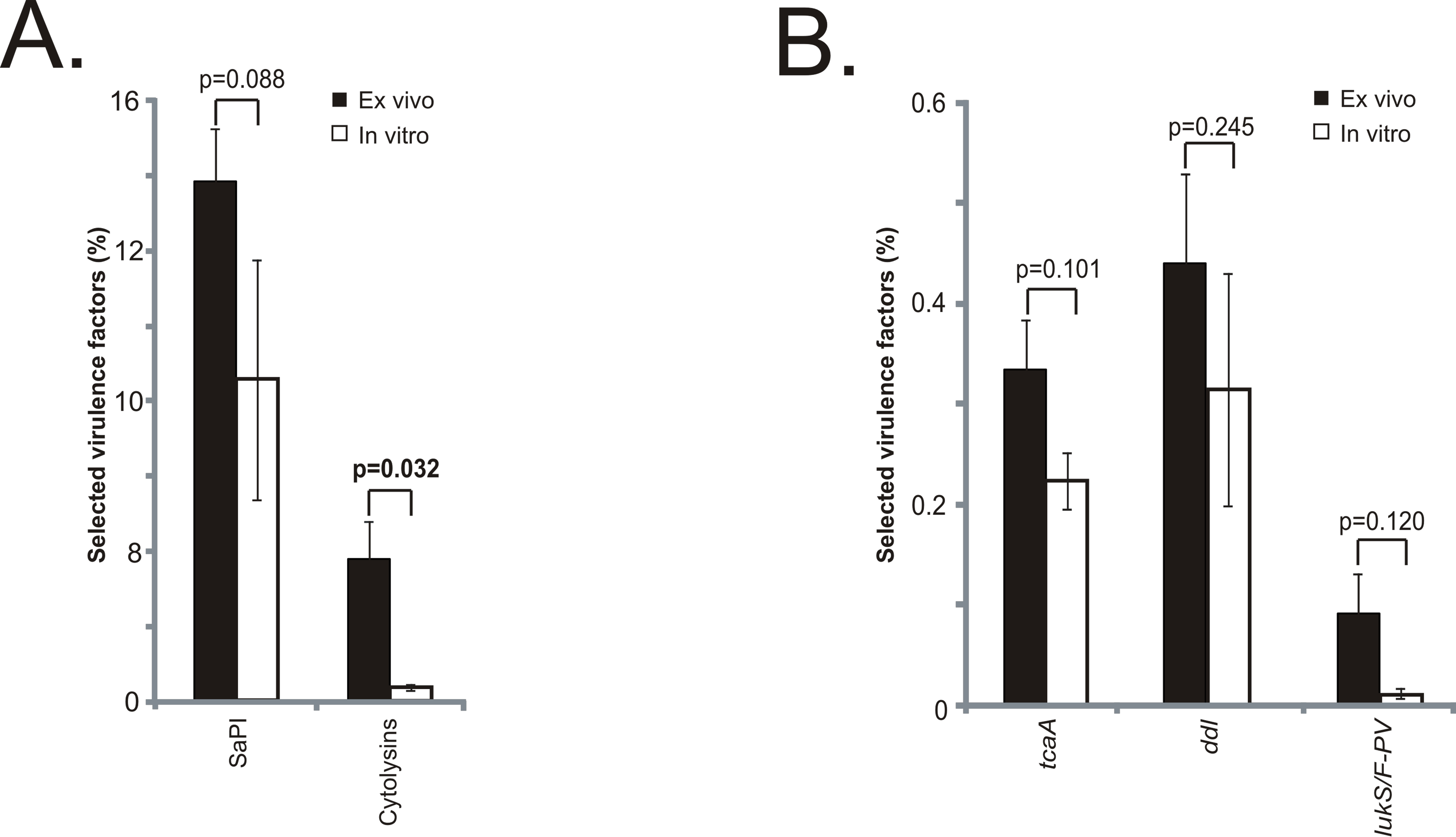

Supplement: Supplemental Figure 5 — Mean percentage (± standard deviation) of the virulence subsystem devoted to individual elements. p-values are shown above each virulence factor. Ex vivo gene expression is represented by black bars and in vitro is represented by white bars. (A) shows Staphylococcal Pathogenicity Islands (SaPIs) and cytolysins, and (B) shows tcaA (teicoplanin-resistance), ddl (involved in cell wall synthesis) and lukS-PV and lukF-PV (encoding Panton-Valentine leukocidin). [file Image5.TIF]
