# Supplementary material for: Identification of staphylococcal phage with reduced transcription in human blood through transcriptome sequencing
Source: Front Microbiol. 2015 Mar 24;6:216. doi: 10.3389/fmicb.2015.00216 (PMC4447126; doi:10.3389/fmicb.2015.00216)
Supplement: Supplementary file 6 [file Table1.DOCX]

| **Subject** | **Antibiotics^a^** | | | | | | | |
| --- | --- | --- | --- | --- | --- | --- | --- | --- |
|  | **Cefazolin** | **Clindamycin^b^** | **Daptomycin** | **Linezolid** | **Oxacillin** | **Rifampin** | **Trimethoprim/**  **Sulfamethaxole** | **Vancomycin** |
| 1MRA | R^c^ | S^d^ | S | S | R | S | S | S |
| 3MRA | R | S | S | S | R | S | S | S |
| 4MRA | R | R | S | S | R | S | S | S |
| 6MRA | R | S | S | S | R | S | S | S |
| 20MRA | R | S | S | S | R | S | S | S |
| 21/23MRA^e^ | R | S | S | S | R | S | S | S |
| 31MRA | R | R | S | S | R | S | S | S |
| 55MRA | R | R | S | S | R | S | S | S |
| 74MRA | R | S | S | S | R | S | S | S |
| 256MRA | R | S | S | S | R | S | S | S |

**Supplemental Table 1**. Antibiotic susceptibilities of MRSA isolates.

^a^Susceptibilities based on Clinical and Laboratory Standards Institute guidelines

^b^Clindamycin susceptibility verified by D-test

^c^Resistant

^d^Susceptible

^e^Subjects 21 and 23 represent the same individual for which samples were collected 48 hours apart
